# Impact of a food supplement containing *Citrus limon* L. Osbeck and *Vitis vinifera* L. extracts, hesperidin and chromium in combination with an isocaloric diet on glucose and lipid metabolism in subjects with impaired fasting blood glucose: a single-center, controlled, randomized, parallel-arm, double-blind clinical trial

**DOI:** 10.3389/fnut.2025.1671102

**Published:** 2025-10-14

**Authors:** Alessandro Di Minno, Maria Vittoria Morone, Daniele Giuseppe Buccato, Lorenza Francesca De Lellis, Hammad Ullah, Luca Borromeo, Angela Cerqua, Roberto Piccinocchi, Agostino Greco, Salvatore Santonastaso, Danaé S. Larsen, Hesham El-Seedi, Costanza Valentina Riccioni, Alessandra Baldi, Gaetano Piccinocchi, Xiang Xiao, Roberto Sacchi, Maria Daglia

**Affiliations:** ^1^Department of Pharmacy, University of Napoli Federico II, Naples, Italy; ^2^CEINGE-Biotecnologie Avanzate, Naples, Italy; ^3^Department of Experimental Medicine, Section of Microbiology and Clinical Microbiology, University of Campania “L. Vanvitelli”, Naples, Italy; ^4^School of Pharmacy, University of Management and Technology, Lahore, Pakistan; ^5^Department of Translational Medical Sciences, University of Naples Federico II, Naples, Italy; ^6^Department of Anaesthesia and Resuscitation A. U. O. Luigi Vanvitelli, Naples, Italy; ^7^Azienda Sanitaria Locale Caserta, Caserta, Italy; ^8^School of Chemical Sciences, The University of Auckland, Auckland, New Zealand; ^9^Department of Chemistry, Faculty of Science, Islamic University of Madinah, Madinah, Saudi Arabia; ^10^R&D Department, Esserre Pharma Srl, Rome, Italy; ^11^Comegen Società Cooperativa Sociale, Naples, Italy; ^12^School of Food and Biological Engineering, Jiangsu University, Zhenjiang, China; ^13^Applied Statistic Unit, Department of Earth and Environmental Sciences, University of Pavia, Pavia, Italy; ^14^International Research Center for Food Nutrition and Safety, Jiangsu University, Zhenjiang, China

**Keywords:** impaired fasting glucose, *Citrus limon* extract, *Vitis viifera* extract, hesperidin, chromium, glucose and lipid metabolism, randomized controlled trial

## Abstract

**Introduction:**

Impaired fasting glycemia (IFG) puts carriers at a significantly higher risk of developing type 2 diabetes and cardiovascular disease. Lifestyle changes, such as increased physical activity and a healthy diet rich in fiber and low in sugar, can often reverse prediabetes and significantly reduce the risk of progressing to type 2 diabetes metabolic disorders, especially diabetes (1).

**Methods:**

This randomized clinical trial aims to evaluate the efficacy and tolerability of a food supplement (FS) containing *Citrus limon* L. Osbeck and *Vitis vinifera* L. extracts, hesperidin from *Citrus sinensis* L. Osbeck, and chromium, in individuals with impaired fasting blood glucose , with the final goal of investigating whether the use of a combination of ingredients, known to exert their positive effects through different mechanisms of action, is a helpful strategy for improving glucose metabolism. Sixty-two subjects (aged 18–75 years) with IFG levels ranging from 100–to 125 mg/dL were assigned to receive either a FS or a placebo (*n* = 31 per group) for 6 months, in combination with an isocaloric diet. Participants took two tablets daily. Biochemical parameters related to glucose and lipid metabolism and markers of liver and kidney functionality were evaluated at baseline (T0), and after 3 and 6 months.

**Results:**

The treatment group showed a significant improvement in fasting glycemia after 3 months, with normalization of glucose levels (*P* < 0.001 vs T0), unlike the placebo group. Statistically improvements were also observed in HDL and LDL cholesterol and triglycerides.

**Discussion:**

Daily intake of the tested FS in combination with an isocaloric diet significantly improved glucose and lipid metabolism, suggesting that the combination of ingredients with different mechanisms of action can be considered a valuable strategy for improving impaired fasting blood glucose and lipid profiles.

**Clinical trial registration:**

www.isrctn.com, ISRCTN15062713.

## 1 Introduction

Updated projections highlight diabetes as a rapidly growing global health emergency, with increasing prevalence driven by factors like urbanization, an aging population, and decreased physical activity. Global or general data from reputable sources (e.g., the World Health Organization or the International Diabetes Federation - IDF), report estimates of Diabetes Mellitus (DM) closer to 463 million people, i.e., about 9–10% of the adult population in 2019–2021. The direct global annual cost of DM is ≥$827 billion, and because of the increased number of people affected, the total global health costs of DM have tripled over the period 2003–2013 (1). In addition, according to the IDF, the number of adults with DM is expected to increase to over 642 million in 2030 and to 783 71 million by 2045.

For 2021 in Italy, where the current study was carried out, the National Institute of Health (ISS) reports a 5.6% a prevalence of DM according to the data from the National Institute of Statistics (ISTAT), corresponding to over 3.5 million people with higher rates in the southern regions.

Such prevalence, with a slowly increasing trend, is age-dependent (≈21% among people over 75) and is lower in the North-West (5.4%), North-East (5.3%) and Center (5.5%) Italian regions, than in the South (7%) and in the islands (6.7%) (2). Moreover, about 1 million Italians with DM have not received a diagnosis so far (3).

Prevention is critical to lower the incidence of DM in the population, with early diagnosis and treatment lowering the risk of long-term complications (4). DM is diagnosed when fasting blood glucose is higher than or equal to 126 mg/dL on two different occasions, while impaired glucose tolerance (IGT) and impaired fasting glucose (IFG) are intermediate conditions (prediabetes) in the transition from normal blood glucose metabolism to DM. Progression to DM in people with prediabetes has been estimated ≈25% over a period of 35 years and 70% over a lifetime period (5). Hence, prevention of abnormal carbohydrate and lipid metabolism is critical to prevent micro- and macrovascular complications due to DM (6). Lifestyle and diet modifications including food supplements are the initial approach in persons with prediabetes, with potential for transition to DM being preventable (7).

High fasting and post-prandial glycemia are risk factors for the development of macrovascular complications in DM (coronary heart disease, and stroke) besides high total blood cholesterol and/or triglycerides, low high-density lipoprotein (HDL) cholesterol, and obesity (6). DM-associated microvascular complications account for chronic kidney disease, retinopathy, and sensory and motor neuropathy (2). Food supplements represent a safe and effective option for reducing the risk factors associated with DM, while minimizing side effects often linked to pharmacological treatments. They can also support better compliance with healthy dietary patterns and lifestyle interventions, including weight management and increased physical activity. Among the bioactive ingredients commonly used in supplements to enhance glucose and lipid metabolism, either alone or in combination, are plant extracts such as *Morus alba* L., *Mangifera indica* L., *Gymnema sylvestre* (Retz.) R. Br. ex Sm., *Cynara scolymus* L., and *Coffea* species (8–12), *Vitis vinifera* L., and *Citrus* fruit, as well as essential trace minerals including zinc, magnesium, vanadium, chromium, and selenium (13, 14).

Chromium has been extensively studied for its role in carbohydrate and lipid metabolism. Chromium deficiency in diet is closely associated with risk factors for diabetes, obesity, and cardiovascular diseases. Chromium activates intracellular signaling pathways, including the translocation of the glucose transporter 4 (GLUT4), which increases the transport of glucose and amino acids. Additionally, chromium inhibits the hepatic enzyme 3-hydroxy-3-methylglutaryl-CoA reductase, interfering with cholesterol metabolism, and enhances insulin sensitivity (15). Chromium supplementation has shown positive effects on glycemic control, improving triglyceride levels and HDL cholesterol (16). Mechanisms of action include increasing the number of insulin receptors, modulating signaling through PI3K, and inhibiting proteins such as PTP1B, which contribute to insulin sensitivity (17).

Hesperidin, a flavonoid found in *Citrus* fruits (i.e., lemons, oranges, and grapefruits) and widely used as a food supplement ingredient, has shown a positive impact on glucose management and insulin resistance. In a 2019 study, hesperidin modulated glucose and fructose transport in Caco-2 cells and improved postprandial glycemic response in healthy volunteers (18). Hesperidin acts by modulating insulin-dependent pathways, such as PI3K/AKT, and improves glucose homeostasis by inhibiting GLUT5. Furthermore, lemon and orange extracts, enriched with hesperidin and other flavonoids, can be beneficial in managing diabetes and obesity by reducing inflammation and improving lipid and glucose metabolism (19).

In addition, flavonoids from *V. vinifera*, such as resveratrol and quercetin, have also shown beneficial effects in improving insulin sensitivity and glycemic control. Clinical studies have suggested that moderate consumption of red wine, rich in polyphenols, is associated with a lower incidence of metabolic syndrome and improvements in glycemic and lipid parameters (20).

Although the individual food supplement ingredients listed above have known effects on glucose and lipid metabolism, it is not known what effects their combination can produce. Thus, we explored the possibility that a food supplement comprised of ingredients allowed under current European regulations, may improve poor individual responses to eating habits and restore the biochemical parameters associated with impaired glucose and lipid metabolism, with the final goal of investigating whether the use of a combination of ingredients, known to exert their positive effects through different mechanisms of action, is a helpful strategy for reducing glycemia and cholesterolemia.

## 2 Materials and methods

### 2.1 Food supplement and placebo

A dietary supplement containing extracts from lemon [*C. limon* (L.) Osbeck, *fructus*] and red grapes (*V. vinifera* L., fructus), hesperidin from *C. sinensis* (L.) Osbeck (*fructus*), and chromium was employed in this trial. The food supplement (FS), is registered with the Italian Ministry of Health following the Legislative Decree 169/2004 and the Guidelines (DGISAN November 2018) that prescribe that clinical trials on foods should focus on food products that fully adhere to the existing food regulations, including notification (notification number 150972) (21). The FS was formulated as coated tablets in compliance with the European regulations on contaminants and microbiological safety standards. It contains a patented complex of flavonoids made of lemon and orange extracts of mediterranean origin, and chromium picolinate. The lemon and red grape extracts are titrated respectively to min. 10% flavonoids (eriocitrin, hesperidin, narirutin) for lemon and 4% anthocyanins for red grape. Excipients include micro-crystalline cellulose as a bulking agent, silicon dioxide, magnesium salts of fatty acids, and cross-linked sodium carboxymethylcellulose as anticaking agents. To ensure blinding, the placebo preparation was indistinguishable in appearance, taste, and packaging from the active treatment. It consisted solely of inert excipients, including microcrystalline cellulose, dicalcium phosphate, magnesium stearate, silicon dioxide, hydroxypropyl cellulose, mono- and diglycerides of fatty acids, yellow iron oxide, and black iron oxide.

### 2.2 Ethical approval, participant consent, and dietary guidelines

The study protocol was approved by the Local Ethics Committee Campania Nord (Protocol number 709, 20 April 2023) and carried out in accordance with the European Union’s Standards of Good Clinical Practice (Directive 2001/20/EEC), the current Declaration of Helsinki concerning medical research on humans (Helsinki 1964, amended by: Tokyo 1975, Venice 1983, Hong Kong 1989, Somerset West 1996, and Edinburgh), and the Guidelines for Good Clinical Practice (CPMP/ICH/135/95). The study was undertaken by a group of general practitioners in Caserta, Italy. To safeguard a clear understanding of the involvement, the participants received oral and written information regarding the study’s objectives, methodology, and procedures. Each participant completed an informed consent form, which included personal data. Each consent was signed both by the participant and the investigator. All were able to understand and willing to sign the informed consent. All participants received guidance and education to follow an isocaloric diet throughout their study. The recommendations provided were based on the Dietary Approaches to Stop Hypertension (DASH) diet, promoted by the National Heart, Lung, and Blood Institute (NHLBI) of the U. S. National Institutes of Health (NIH) to prevent/manage high blood pressure. Beyond its benefits for blood pressure regulation and prevention of obesity, the DASH diet also improves insulin resistance, and hyperlipidemia (22). The study protocol did not allow additional dietary supplements, functional foods, or pharmacological treatments aimed at reducing blood glucose levels, lipids, or body weight. The clinical data of individual participants was handled anonymously in accordance with current privacy regulations.

### 2.3 Clinical trial design

This single-center, randomized, controlled, parallel-group double-blinded (both for the principal investigator and the enrolled subjects) clinical study tested the efficacy of the dietary supplement to maintain normal carbohydrate metabolism and lower plasma glucose levels in subjects with impaired fasting glycemia (100–125 mg/dL) after three and 6 months of treatment. Nor was the treatment assignment disclosed to the sponsor, or any other individuals involved in the study, except in the event of an emergency. The study design included two experimental groups (31 subjects for each group): the treatment group in which two tablets of the food supplement were administered daily (after lunch and dinner) and the placebo group consisting of subjects who took two tablets a day (after lunch and dinner) of the placebo. The food supplement daily dose contains 250 μg of chromium and 560 mg of flavonoids: hesperidin 480 mg, eriocitrin 75 mg, and other flavonoids 5 mg (including malvidin 3-O-glycoside, delphinidin 3-O-glycoside, petunidin 3-O-glycoside, cyanidin 3-O-glycoside, quercetin, quercetin 3-O-glycoside, kaempferol 3-O-rutinoside, and resveratrol). The randomization sequence was generated by a statistician using STATA 16 software (Stata Statistical Software: Release 16. College Station, TX: StataCorp LLC), and the subjects were randomly and unpredictably assigned into one of the two treatment groups (dietary supplement or placebo) using a 1:1 ratio allocation (simple randomization). This procedure minimized selection bias, preventing systematic differences between baseline characteristics of the groups (prognostic and treatment response imbalance).

### 2.4 Participants and recruitment

In their practice, general practitioners (GPs) are in close contact with patients. By integrating daily work with clinical research, they contribute to the development of diagnostic treatments, and methods in a real-world care setting. The clinician who wrote the protocol, selected the patients and followed them up during the study, carried out preliminary meetings to publicize the study among GPs in Caserta area interested in clinical research. Both for patients and GPs who referred their patients (but did not directly participate in the trial), the study information was shared in Italian. Among interested patients, updated individual medical records were employed by each GP to identify potential study participants. For each patient, the time committed to the study was limited to the time needed for regular blood tests to assess efficacy, safety and potential toxicity of the supplement (i.e., at baseline, after 90, and after 180 days of treatment). Participants were recruited based on their clinical history and the information gathered during the medical check-up carried out as part of the screening visit. Indeed, in the enrollment phase, the subjects were analyzed to confirm the diagnosis of impaired fasting glucose (IFG) control (glucose levels ranging 100–125 mg/dL) (23). Eligible subjects were non-diabetic individuals aged 18–75 years with impaired fasting glucose (IFG). All had an average LDL cholesterol value ≤130 mg/dL. Exclusion criteria were individuals <18 or >75 years of age, those at high cardiovascular risk according to the Progetto Cuore of the Istituto Superiore di Sanità (24), and those classified as obese by the Italian Ministry of Health (body mass index, BMI ≥ 30). Progetto CUORE is managed by the Italian National Institute of Health (ISS). Its objectives are to monitor risk factors and non-communicable diseases in the Italian population to support the National and Regional Prevention Plans, evaluate the effectiveness of public health campaigns, develop tools for cardiovascular risk assessment, and contribute to international health knowledge. This is achieved through periodic, population-based Health Examination Surveys (HES) and a national registry for major fatal and non-fatal cardiovascular events (25).

Subjects undergoing pharmacological treatment for DM, even at low doses, or those who had taken blood glucose regulating supplements in the 2 weeks before enrollment, blood donors within the 3 months before recruitment, individuals with limited self-sufficiency, or those unable to comply with the study protocol were also excluded. Pregnant or breastfeeding women, as well as those planning a pregnancy, were not eligible for the study as were those who were unable to attend scheduled visits or were deemed unsuitable by the principal investigator due to other medical conditions. Further exclusion criteria included a history of allergies to any of the ingredients in the dietary supplement or in the placebo, substance abuse (including drugs or alcohol), and smoking, including the use of electronic nicotine devices.

### 2.5 Outcomes of the study

The primary outcome of the study was to evaluate the clinical efficacy of the food supplement in combination with an *ad hoc* diet to maintain normal carbohydrate metabolism by reducing fasting plasma glucose levels in individuals with impaired fasting glucose. Secondary outcomes were evaluated to explore the metabolic (and potential toxic) effects of the food supplement. Glycosylated hemoglobin (HbA1c) levels a reliable index of blood glucose levels in the three preceding months, fasting blood glucose (FBG), fasting insulin (INS) concentrations, and insulin resistance using the homeostatic model assessment of insulin resistance (HOMA-IR) index were employed to investigate potential benefits of the dietary supplement on glucose metabolism. The effects on lipid metabolism were examined by measuring total cholesterol (TC), low-density lipoprotein cholesterol (LDL-C), high-density lipoprotein cholesterol (HDL-C), and plasma triglycerides (TG). Body weight regulation was also considered among secondary outcomes, and changes in BMI, HOMA-IR, blood pressure, [systolic (SBP) and diastolic (DBP)], and waist circumference (WC) were measured at each time point over the course of the study. Additionally, the study aimed to evaluate the impact of the supplement on systemic inflammation by determining white blood cell counts (WBC), erythrocyte sedimentation rate (ESR), and C-reactive protein (CRP). At baseline (T0), after 90 days (T1), and after 180 days (T2), blood samples were collected from the recruited subjects to evaluate such parameters. The total duration of the study was 12 months, including 2 months for patient enrollment, 6 months for treatment and monitoring, and 4 months for data collection and analysis. All secondary outcomes were assessed at the same time points as the primary outcome [baseline (T0), after 90 days (T1), and after 180 days (T2) of treatment].

### 2.6 Data collection

Data collection was carried out using specific Case Reporting Forms (CRFs) divided into two main sections. The first section recorded personal data, patient medical history, concomitant medication use, and treatment group assignment at the time of enrollment. The second section included the results of analyses performed on the blood samples collected from the enrolled subjects.

### 2.7 Safety and tolerability

Participants were monitored throughout the study and underwent regular blood tests to assess potential toxicity. To assess the safety profile of the supplement, potential liver and renal toxicity was monitored through the measurements of aspartate aminotransferase (AST), alanine aminotransferase (ALT), and creatinine (CRE) levels at baseline (T0), after 90 (T1), and after 180 days (T2) of treatment.

### 2.8 Statistical analysis

Power analysis, carried out on the interaction between measurement time points and treatment group to verify whether the difference between the placebo (CTRL) and treatment (TRAT) groups varied over time, considered three levels of statistical power (1-*β* = 0.80, 0.95, and 0.99), a significance level (*α*) of 0.05, and three levels effect sizes (Cohen’s *f* = 0.10, 0.25 and 0.40). Based on these parameters, the minimum required sample size was determined to be 54 subjects, with an additional 15% added to account for potential dropout, resulting in a total of 62 participants (31 per group).

The most appropriate statistical approach for this study was a random interception linear mixed model (LMM) analysis of variance, with primary and secondary outcomes as the dependent variable. The model included measurement time points (T0, T1, and T2), treatment group and their interaction as fixed factors, individual patient identity being used as a random factor to account for inter-individual variability in treatment responses. For each measurement in both experimental groups, a descriptive analysis was first performed, calculating the mean, standard deviation, and min-max range for all response variables under investigation. Patient identity was treated as a random factor to account for inter-individual variability, thereby increasing the power and accuracy of the model. Independent analyses were conducted for each response variable. All data were entered into a dedicated database, and statistical analyses were performed by a biostatistician blinded to treatment allocation. The lme4 (26) package in R ver. 4.4 (27) was used for the analysis.

## 3 Results

**Figure 1:**
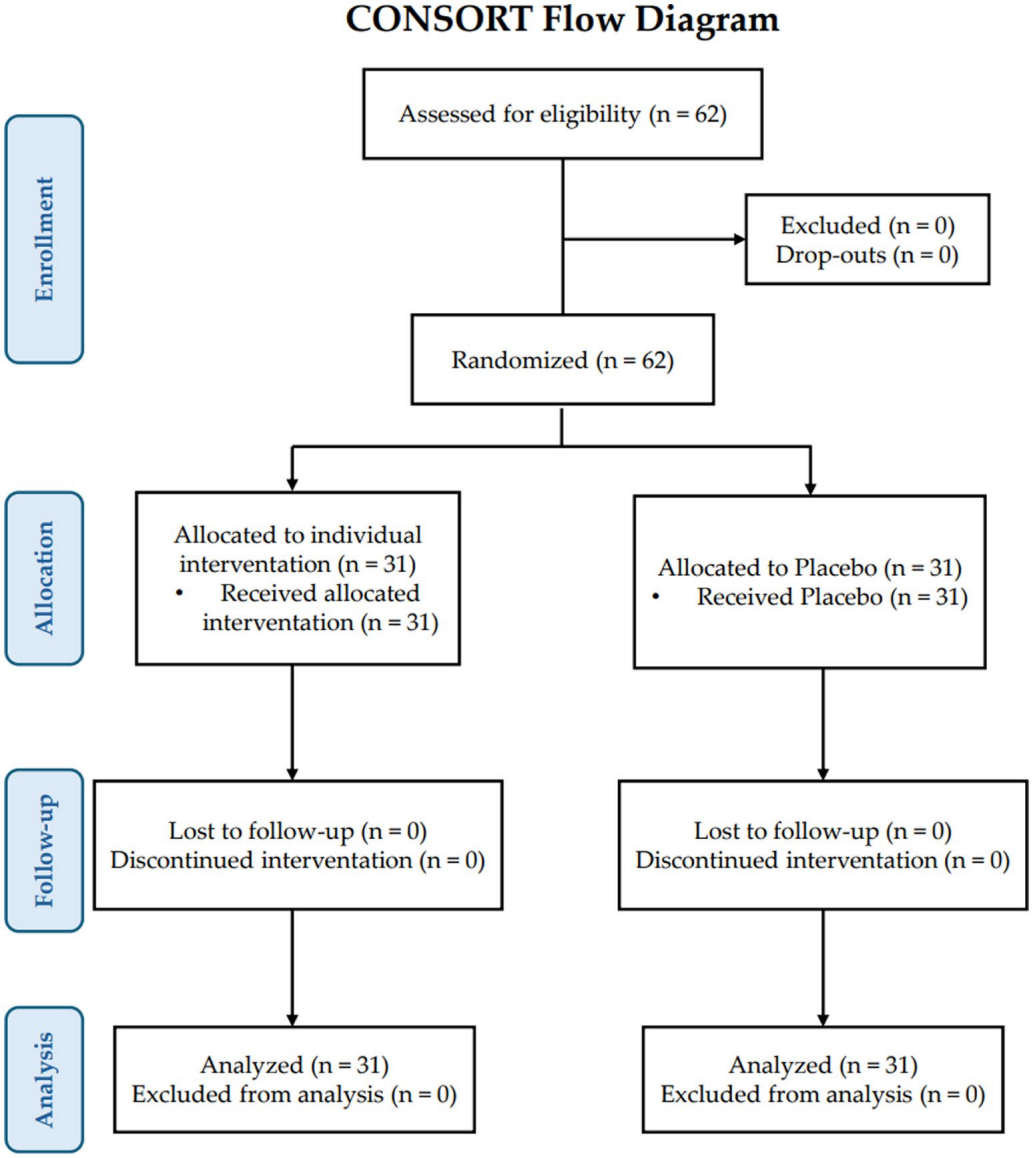
Consort PRO flow diagram.

The study flow chart, prepared following the CONSORT PRO reporting guidelines (28), is presented in Figure 1. Descriptive statistics (mean, standard deviation, and range of values) for the 18 variables measured at T0, T1, and T2 in the placebo and the treatment groups are reported in Table 1. LMM analysis of variance of individual data analysis is reported in Tables 2, 3 and illustrated in Supplementary Figures S2–S7.

**Table 1:** Descriptive statistics (mean, standard deviation, and range of values) for the 18 variables measured at time points T0, T1, and T2 in the placebo and treatment groups.

Variable	Placebo group			Treatment group		
	T0	T1	T2	T0	T1	T2
Age	42.19 ± 18.26			44.06 ± 15.92		
Sex	12 Male; 19 Female			17 Male; 14 Female		
FBG (mg/dl)	113 ± 8	111 ± 6	107 ± 8	114 ± 5	95 ± 7	94 ± 6
	(100–125)	(101–123)	(95–124)	(105–123)	(85–107)	(85–115)
HbA1c (%)	5 ± 1	5 ± 1	5 ± 1	5 ± 1	4 ± 1	4 ± 1
	(4–6)	(4–6)	(4–6)	(4–6)	(4–5)	(4–5)
INS (pmol/L)	14 ± 7	14 ± 6	11 ± 5	14 ± 7	14 ± 5	12 ± 3
	(5–24)	(4–23)	(4–20)	(4–24)	(4–20)	(8–22)
HOMA-IR	4 ± 2	4 ± 2	3 ± 1	4 ± 2	3 ± 1	3 ± 1
	(2–7)	(2–15)	(1–5)	(1–7)	(1–5)	(2–6)
TC (mg/dL)	167 ± 34	164 ± 28	162 ± 9	173 ± 28	149 ± 27	147 ± 19
	(43–220)	(111–218)	(112–213)	(131–220)	(103–196)	(113–179)
HDL-C (mg/dL)	43 ± 14	45.1 ± 11.9	47 ± 11	38 ± 11	44 ± 8	56 ± 12
	(20–60)	(26–60)	(21–60)	(20–59)	(32–58)	(37–74)
LDL-C (mg/dL)	118 ± 25	108 ± 25	113 ± 20	114 ± 33	87 ± 29	73 ± 22
	(87–154)	(77–147)	(83–156)	(58–171)	(39–142)	(24–112)
TG (mg/dL)	93 ± 24	99 ± 25	112 ± 21	107 ± 27	92 ± 23	93 ± 17
	(57–139)	(62–130)	(57–149)	(60–150)	(50–130)	(59–116)
BMI (kg/m^2^)	22 ± 4	23 ± 3	22 ± 3	23 ± 4	23 ± 3	23 ± 3
	(16–30)	(18–30)	(17–30)	(18–29)	(17–27)	(18–26)
WC (cm)	98 ± 5	98 ± 4	99 ± 5	99 ± 5	99 ± 5	99 ± 4
	(88–105)	(90–105)	(90–113)	(90–108)	(90–108)	(91–107)
DBP (mmHg)	71 ± 6	79 ± 8	77 ± 9	76 ± 8	78 ± 9	75 ± 9
	(63–85)	(60–88)	(62–88)	(60–90)	(60–89)	(60–90)
SBP (mmHg)	129 ± 18	121 ± 18	121 ± 18	129 ± 22	129 ± 12.	122 ± 14
	(106–156)	(82–148)	(99–160)	(100–160)	(104–149)	(100–147)
WBC (10^3^/μL)	7 ± 2	7 ± 2	7 ± 2	8 ± 2	7 ± 2	8 ± 2
	(3–10)	(3–10)	(3–10)	(4–11)	(4–11)	(5–10)
ESR (mm/h)	15 ± 6	13 ± 6	12 ± 8	17 ± 5	15 ± 5	16 ± 6
	(5–25)	(2–24)	(3–24)	(6–25)	(6–25)	(5–25)
CRP (mg/L)	4 ± 2	4 ± 2	4 ± 2	4 ± 1	5 ± 1	4 ± 2
	(1–7)	(1–7)	(1–7)	(2–7)	(2–7)	(2–7)
CRE (mg/dL)	1.1 ± 0.2	0.9 ± 0.2	1.1 ± 0.2	1.1 ± 0.1	1.1 ± 0.1	1.1 ± 0.1
	(0.7–1.3)	(0.7–1.3)	(0.7–1.3)	(0.7–1.3)	(0.7–1.3)	(0.7–1.3)
AST (U/L)	25 ± 9	24 ± 10	24 ± 10	19 ± 9	22 ± 9	17 ± 9
	(12–46)	(10–47)	(7–45)	(0–36)	(5–36)	(5–37)
ALT (U/L)	23 ± 10	21 ± 14	29 ± 14	27 ± 10	29 ± 13	28 ± 12
	(10–47)	(7–47)	(8–48)	(5–42)	(6–45)	(6–45)

**Table 2:** Comparison between T0, T1 and T2 in the efficacy variables evaluated to explore the metabolic effects of the supplement: linear mixed models (LMM).

Variable	Parameters	df	*F*	*p*
FBG (mg/dL)	Measure	2,123	73.13	**<0.001**
	Treatment	1,56	61.48	**<0.001**
	Sex	1,63	0.001	0.97
	Age	1,59	0.736	0.39
	Size x Treatment	2,123	35.02	**<0.001**
HbA1c (%)	Measure	2,123	8.189	**<0.001**
	Treatment	1,53	19.61	**<0.001**
	Sex	1,60	9.898	**0.0026**
	Age	1,58	1.729	0.19
	Size x Treatment	2,123	0.461	0.63
INS (pmol/L)	Measure	2,123	3.776	**0.026**
	Treatment	1,54	0.001	0.98
	Sex	1,61	5.150	**0.027**
	Age	1,58	6.447	**0.014**
	Size x Treatment	2,123	0.115	0.89
HOMA-IR	Measure	2,124	6.506	**0.0020**
	Treatment	1,53	3.908	0.053
	Sex	1,60	7.906	**0.0066**
	Age	1,58	11.71	**0.0011**
	Size x Treatment	2,124	2.012	0.14
TC (mg/dL)	Measure	2,119	5.536	**0.0050**
	Treatment	1,47	2.706	0.11
	Sex	1,54	1.411	0.24
	Age	1,52	0.668	0.42
	Size x Treatment	2,119	3.133	**0.047**
HDL-C (mg/dL)	Measure	2,124	18.14	**<0.001**
	Treatment	1,54	1.228	0.27
	Sex	1,61	13.02	**<0.001**
	Age	1,58	3.253	0.08
	Size x Treatment	2,124	7.762	**<0.001**
LDL-C (mg/dL)	Measure	2,178	13.55	**<0.001**
	Treatment	1,178	31.35	**<0.001**
	Sex	1,178	0.008	0.93
	Age	1,178	0.116	0.73
	Size x Treatment	1,178	7.138	**0.0010**
TG (mg/dL)	Measure	2,121	1.513	0.22
	Treatment	1,49	1.563	0.22
	Sex	1,56	0.164	0.69
	Age	1,54	4.344	**0.042**
	Size x Treatment	2,121	8.751	**<0.001**
BMI (kg/m^2^)	Measure	2,121	0.506	0.60
	Treatment	1,72	2.091	0.15
	Sex	1,90	10.64	**0.0015**
	Age	1,80	0.892	0.35
	Size x Treatment	2,121	2.343	0.10
WC (cm)	Measure	2,106	0.705	0.50
	Treatment	1,65	7.552	**0.0077**
	Sex	1,84	2.819	0.10
	Age	1,77	0.129	0.72
	Size x Treatment	2,106	0.533	0.59
DBP (mmHg)	Measure	2,178	5.805	**0.0036**
	Treatment	1,178	0.240	0.62
	Sex	1,178	0.000	0.99
	Age	1,178	4.143	**0.043**
	Size x Treatment	2,178	3.459	**0.033**
SBP (mmHg)	Measure	2,128	3.241	**0.042**
	Treatment	1,58	0.923	0.34
	Sex	1,65	0.271	0.60
	Age	1,63	0.403	0.53
	Size x Treatment	2,128	1.123	0.33

The bold values indicated significance of results.

**Table 3:** Comparison between T0, T1 and T2 in the safety variables evaluated to explore potential toxic effects of the supplement: linear mixed models (LMM).

Variable	Parameters	df	*F*	*p*
WBC (10^3^/μL)	Measure	2,178	0.525	0.59
	Treatment	1,178	13.46	**<0.001**
	Sex	1,178	4.236	**0.041**
	Age	1,178	0.292	0.59
	Size x Treatment	2,178	0.652	0.52
ESR (mm/h)	Measure	2,178	1.732	0.18
	Treatment	1,178	7.984	**0.0053**
	Sex	1,178	1.881	0.17
	Age	1,178	1.216	0.27
	Size x Treatment	2,178	0.926	0.40
CRP (mg/L)	Measure	2,178	1.467	0.23
	Treatment	1,178	1.782	0.18
	Sex	1,178	0.143	0.71
	Age	1,178	0.064	0.80
	Size x Treatment	2,178	1.384	0.25
CRE (mg/L)	Measure	2,120	3.283	**0.041**
	Treatment	1,49	10.17	**<0.001**
	Sex	1,56	3.008	0.09
	Age	1,54	6.529	**0.013**
	Size x Treatment	2,120	9.827	**<0.001**
AST (U/L)	Measure	2,178	0.724	0.49
	Treatment	1,178	14.86	**<0.001**
	Sex	1,178	0.202	0.65
	Age	1,178	0.268	0.61
	Size x Treatment	2,178	1.108	0.33
ALT (U/L)	Measure	2,178	1.739	0.18
	Treatment	1,178	3.824	0.052
	Sex	1,178	0.059	0.81
	Age	1,178	5.123	**0.025**
	Size x Treatment	2,178	2.083	0.13

The bold values indicated significance of results.

### 3.1 Blood glucose levels

The LMM model for blood glucose levels identified a significant effect of measurement (*p* < 0.001), treatment (*p* < 0.001), and their interaction (*p* < 0.001), with no significant effects of age and sex (Table 2). These findings indicate that blood glucose changed differently over time between the two groups (Supplementary Figure S2). Blood glucose did not differ between groups at baseline (T0, *p* = 0.66). In the placebo group, blood glucose significantly decreased from T0 to T2 (*β* = 5.39 ± 1.58, t₁₂₃ = 3.409, *p* < 0.001). However, this reduction was not clinically relevant, as glucose levels stayed within the pre-diabetic range (100–125 mg/dL). Conversely, in the treatment group, blood glucose levels reached normal fasting levels already at T1 measurements (T0 vs. T1: *β* = 18.9 ± 1.58, t_123_ = 11.94, p < 0.001), to remain unchanged between the T1 and T2 (*β* = 1.29 ± 1.58, t_123_ = 0.816, *p* = 0.42). Thus, the decrease between T0 and T2 measurements was significant (*β* = 20.2 ± 1.58, t_123_ = 12.76, *p* < 0.001) and clinically relevant and normal fasting blood glucose values were restored after the food supplement treatment.

### 3.2 HbA1c, insulin and HOMA-IR responses

The LMM model for HbA1c (Table 2) identified significant effects for measurement (*p* < 0.001) and treatment (*p* < 0.001). However, the non-significant interaction between measurement and treatment (*p* = 0.63) suggests that the pattern of change over time was similar in both groups. In the model, sex had a significant effect (*p* = 0.0026), while age did not. At baseline (T0) the HbA1c levels were slightly higher in the treatment group (*β* = 0.31 ± 0.15, t_171_ = 2.044, *p* = 0.042). HbA1c levels significantly decreased between T0 and T1 in both groups (Table 1). In contrast, the overall T0-T2 reduction was significant only in the treatment group (*β* = 0.43 ± 0.14, t₁₂₃ = 2.971, *p* = 0.0036). Moreover (Supplementary Figure S3), at all time points, HbA1c levels were lower in the treatment than in the placebo group (*p* < 0.042). Regardless of the experimental group and measurement, HbA1c was significantly higher in men (*β* = 0.30 ± 0.10, t_60_ = 3.164, *p* = 0.0026). Insulin levels fluctuated over time in both groups (Supplementary Figure S3), with a significant effect of measure (*p* = 0.026) but not of treatment (*p* = 0.98) or their interaction (*p* = 0.89). Sex (*p* = 0.027) and age (*p* = 0.014) had a significant impact: insulin levels increased with age (*β* = 1.11 ± 0.44, t_58_ = 2.539, *p* = 0.014) and were higher in men than in women (*β* = 2.00 ± 0.88, t_60_ = 2.269, p = 0.027). In the placebo group, insulin levels decreased between T1 and T2 (*β* = 2.74 ± 1.29, t_123_ = 2.126, *p* = 0.035), but the overall T0 to T2 reduction was not significant (*p* = 0.078); a similar trend was observed in the treatment group (*p* > 0.13). At all times, no difference was found between the two groups (*p* > 0.69). HOMA-IR index behaved similarly (Table 1). Indeed, the LMM model identified an effect for measurement (*p* = 0.0020) but not for treatment (*p* = 0.053) or their interaction (*p* = 0.149). A significant effect was also found for sex and age: values increased with age (*β* = 0.41 ± 0.12, t_58_ = 3.422, *p* = 0.0011) and were higher in men than in women (*β* = 0.70 ± 0.25, t_60_ = 2.812, *p* = 0.0066). At variance with the placebo group, (*p* = 0.070; Table 1), the HOMA index drop between T0 and T2 in the treatment group, was statistically significant (*β* = 1.01 ± 0.39, t₁₂₃ = 2.601, *p* = 0.010).

### 3.3 Impact of the supplement on lipid profile

In the placebo group, TC levels were stable across all time points, with no changes between T0 and T2 (*p* > 0.49). Conversely, in the treatment group, TC levels significantly decreased between T0 and T1 (*p* < 0.001) and remained stable between T1 and T2 (*p* = 0.83). As a result, the overall reduction from T0 to T2 was statistically significant (*p* < 0.001; Table 1; Supplementary Figure S4). On the other hand, at baseline (T0), there was no difference in TC levels between the two groups (*p* = 0.30) while at T1 and T2, TC levels were lower in the treatment than in the placebo group, the difference being only significant at T1 comparison (*β* = 14.8 ± 7.17, t_175_ = 2.066, *p* = 0.040; Table 2). This suggests that the food supplement reduced TC levels early in the study, with a sustained effect over time. HDL values did not differ between the two groups at baseline (T0) and T1 (*p* > 0.15). In subjects who ingested the placebo, HDL values did not change between T0 and T2 (*p* > 0.13). In contrast, in subjects ingesting the food supplement, they increased significantly over time, both between T0 and T1 (*β* = 5.87 ± 2.63, t_123_ = 2.229, *p* = 0.028) and between T1 and T2 (*β* = 12.3 ± 2.63, t_123_ = 4.667, *p* < 0.001). Hence, the difference between T0 and T2 was significant (*β* = 18.2 ± 2.63, t_123_ = 6.896, *p* < 0.001; Table 2; Supplementary Figure S4). In addition, those ingesting the supplement had higher HDL levels than the control group at T2 (*p* < 0.001). Regardless of the placebo or food supplement, women had consistently higher HDL values than men (*β* = 6.52 ± 1.81, t_61_ = 3.609, *p* < 0.001). The analysis of LDL values revealed a significant effect of measurement (*p* < 0.001), treatment (*p* < 0.001), and their interaction (*p* = 0.0010), but not for sex and age (Table 2). There were no significant differences in LDL values between the subjects ingesting the placebo and those ingesting the food supplement at baseline (*p* = 0.53, Table 1; Supplementary Figure S4). The LMM model showed that LDL values vary differently between the two groups as measurements progressed (Supplementary Figure S4). In the control group, LDL values did not change between T0 and T2 (*p* > 0.14). In contrast, in subjects who ingested the supplement, LDL values decreased over time, and the difference between T0 and T2 was significant (*β* = 41.1 ± 6.69, t_178_ = 6.153, *p* < 0.001). At T1 measurement, LDL values were significantly lower in subjects who ingested the supplement compared to those who ingested the placebo (*β* = 21.6 ± 6.72, t_178_ = 3.216, *p* = 0.0015), and this difference increased at the T2 measurement (*β* = 39.9 ± 6.72, t_178_ = 5.945, *p* < 0.001). Analysis of TG data identified a significant effect for the interaction between measurement and treatment (*p* < 0.001), but not for the main effects of measurement (*p* = 0.22) and treatment (*p* = 0.22). A significant effect was also found for age (*p* = 0.042) but not for sex (Table 2). TG values changed over time in opposite directions in the experimental and the control group (Supplementary Figure S4). In the placebo group, TG levels increased between T0 and T2 (*p* = 0.0012), while, in the supplement group, they decreased between T0 and T1 (*p* = 0.0096) and persisted as stable thereafter (*p* = 0.95). The overall decrease from T0 to T2 in the treatment group was significant (*p* = 0.011). Finally, at baseline (T0), TG levels were higher in the treatment than in the placebo group (*β* = 13.4 ± 5.83, t_174_ = 2.309, *p* = 0.022), while at T2, TG levels in the placebo group were higher than those of the supplement group (*p* < 0.001). Regardless of the experimental group and measurement, TG values increased with the age of the subjects (*β* = 3.68 ± 1.77, t_54_ = 2.084, *p* = 0.042).

### 3.4 Effects on anthropometric measurements and on blood pressure

There were no significant effects for measurement, treatment, or their interaction for BMI measurements (Table 3). Accordingly, there is no difference in BMI values between the two groups over time (Table 1; Supplementary Figure S5). Regardless of the experimental group and measurement, sex (but not age) had a significant effect (*p* = 0.0015), women having higher BMI values (*p* = 0.0015) than men. The LMM model revealed a significant treatment effect on WC (*p* = 0.0077; Table 2). However, WC did not change across sequential measurements, nor were significant effects found for measurement, interaction, age, or sex. Both in the placebo and the treatment group (*p* > 0.19 and *p* > 0.55, respectively), WC values were stable over time. The LMM model for DBP identified a significant effect for measurement (*p* = 0.0036) and for measurement × treatment interaction (*p* = 0.0033), but not for the main effect of treatment (*p* = 0.62). A significant effect for the age (*p* = 0.043) but not for sex also emerged (Table 3). DBP values changed differently between the two experimental groups as measurements progress. In subjects ingesting the placebo, DBP values increased significantly between T0 and T1 (*β* = 8.10 ± 2.08, t_178_ = 3.898, *p* < 0.001) and then persisted unchanged; hence, a significant difference between baseline and T2 was detected (*β* = 5.87 ± 2.08, t_178_ = 2.827, *p* = 0.0052). In the treatment group, DBP values did not change between T0 and T2 (*p* > 0.13). Regardless of the experimental group and measurement, DBP significantly increases with age (*β* = 1.24 ± 0.61, t_178_ = 2.036, *p* = 0.043). A significant effect in SBP was identified for measurement (*p* = 0.042), but not for treatment or their interaction. Nor were their significant effects for age and sex of the subjects (Table 2). Despite fluctuations between repeated measurements, the model showed that SBP does not vary between the two groups. In subjects on placebo, SBP values showed slight fluctuations between T0 and T2 (*p* > 0.056). The same was observed in subjects who ingested the supplement (*p* > 0.082). At all time points, SBP values did not differ between the two groups (*p* > 0.080).

### 3.5 Safety assessment: systemic inflammatory markers

The LMM model showed that WBC counts were significantly affected by treatment (*p* < 0.001) and sex (*p* = 0.041), but not by measurement (*p* = 0.59), measurement × treatment interaction (*p* = 0.52), or age (Table 3). At baseline (T0) the WBC count was higher in the placebo group (*β* = 1.30 ± 0.51, t_178_ = 2.621, *p* = 0.0095). WBC count did not change between T0 and T2 in subjects who received the food supplement (*p* > 0.15), or the placebo (*p* > 0.73). The WBC count was substantially higher in men than in women (*β* = 0.61 ± 0.30, t_178_ = 2.058, *p* = 0.041) regardless of the experimental group and measurement period. The LMM model for ESR values revealed a significant effect only for treatment (*p* = 0.0053). Thus, the model indicated that ESR values differed between experimental groups but do not vary between consecutive measurements within each experimental group. Indeed, in subjects who ingested placebo, the value did not change between repeated measurements or between T0 and T2 (*p* > 0.056). The same is true for subjects who ingested the supplement (*p* > 0.21). Finally, the ESR value was significantly lower in the control group only at the T2 measurement (T0 vsT2: *β* = 4.32 ± 1.57, t_178_ = 2.747, *p* = 0.0066).

### 3.6 Safety assessment: liver and kidney biomarkers

The LMM model for creatinine (Table 3) identified a significant effect for measurement (*p* = 0.041), treatment (*p* < 0.001), interaction (*p* < 0.001) and age (*p* = 0.013) but not for the sex. The model indicated that the creatinine value varied differently in the two experimental groups as measurements progressed (Supplementary Figure S7). At baseline (T0) creatinine values did not differ between groups (*p* = 0.95). In subjects on placebo as well as for those ingesting the supplement, there was no significant difference between the creatinine values at T0 and T2 (*p* = 0.94 and *p* = 0.58, respectively). At T1 measurement, those who ingested the supplement had creatinine values higher than those in the control group (*β* = 0.23 ± 0.04, t_172_ = 5.440, *p* < 0.001). Regardless of the experimental group and measurement, the creatinine value decreased significantly with the age of the subjects (*β* = 0.03 ± 0.01, t_54_ = 2.555, *p* = 0.013). The LMM model for AST levels identified a significant effect only for treatment (*p* < 0.00). AST levels differed between experimental groups but were stable at all time points within each group (Table 1). In the placebo group, AST levels did not change between T0 and T2 (*p* > 0.69), while they slightly but not significantly decreased at T2 in the treatment group (*p* > 0.064; Table 1; Supplementary Figure S7). Notably, AST levels were lower in the treatment group, with statistically significant differences at baseline (T0, *β* = 6.03 ± 2.38, t_178_ = 2.535, *p* = 0.012) and T2 (*β* = 7.42 ± 2.38, t_178_ = 3.119, *p* = 0.0021). The LMM model for ALT levels did not identify a significant effect for measurement, treatment, or their interaction. A significant effect was found for age (*p* = 0.025), but not for sex. Consequently, ALT levels did not differ between the two groups and did not change over time (Table 1; Supplementary Figure S7). Regardless of the experimental group and measurement time, ALT levels significantly decreased with increasing age (*β* = 2.06 ± 0.91, t_178_ = 2.263, *p* = 0.025).

## 4 Discussion

The present study documents the beneficial effect on glucose and lipid metabolism of the combination of different ingredients permitted under the current European regulations on food supplements i.e., extracts from lemon [*Citrus limon* (L.) Osbeck, fructus] and red grapes (*Vitis vinifera L*., fructus), as well as hesperidin from *Citrus sinensis* (L.). Osbeck (fructus) and chromium, providing the rationale for a strategy based on the use of food supplement ingredients with different mechanisms of action to improve impaired glucose metabolism and poor individual responses to eating habits, and delay the use of drugs in subjects with borderline glycemia (114 mg/dL). At T2, subjects receiving the isocaloric diet and placebo showed a reduction in fasting blood glucose levels to 107 mg/dL, which nonetheless remained above the normal threshold of 100 mg/dL. In combination with the *ad hoc* diet, the food supplement normalized fasting blood glucose values (94 mg/dL, *p* < 0.001) as early as after 3-mo intake and kept significantly lower than in the placebo group after 6-mo intake. At all time points, HbA1c levels were lower in the treatment group than in the placebo group (*p* < 0.042), as was found for the HOMA index (*p* = 0.010). In the treatment group, TC levels significantly decreased between T0 and T1 (*p* < 0.001) and remained stable between T1 and T2 (*p* = 0.83). Triglyceride levels behaved similarly (*p* = 0.011). In parallel, HDL values increased significantly in subjects ingesting the supplement and, at variance with the control group (*p* > 0.14), LDL values decreased significantly between T0 and T2 (*p* < 0.001) in subjects who ingested the food supplement. In both groups, there was no effect of the diet with/without the food supplement on BMI, WC, SBP, and DBP. WBC counts did not change over time both in subjects who received the supplement (*p* > 0.15), and the placebo (*p* > 0.73), as did ESR, CRP, creatinine levels AST and ALT values.

The relevance of these data with respect to the current understanding of nutrition, metabolism and cardiovascular disease deserves comment. Values below 2.6 of the HOMA index are considered normal when quantifying insulin resistance and in turn the pancreatic beta cell function (29). Both in the treatment and in the placebo group, the index decreased to an average value of 2.9 in the treatment and 3.0 in the placebo group. The absence of a significant difference between the two groups even though there is a decrease in blood sugar in the group that received the dietary supplement, is likely related to the fact that lifestyle changes (e.g., diet) per se improve insulin resistance (7). Impaired glucose tolerance and DM are different clinical entities. Both the treatment and the control group of this study included subjects with impaired fasting blood glucose and glycated hemoglobin (H1bAc) levels in the normal range (<5.7%). H1bAc reflects the average blood glucose levels in the last 3 months (30), thus providing a reliable overview of individual glycemic control. The absence of statistical significance between the H1bAc values of the treatment and the control groups is in keeping with the careful selection of the volunteers in this study. Confirmation of the concept that the individuals selected for this study only have an impaired glucose tolerance, circulating insulin levels that range from 4 to 24 *μ*-units/ml of blood under physiological conditions, did not differ between the placebo and the treatment group. According to Progetto Cuore, optimal TC should not exceed 200 mg/dL; optimal LDL-cholesterol values should not exceed 100 mg/dL; optimal triglyceride values should not exceed 150 mg/dL, and optimal HDL-cholesterol values should be ≥50 mg/dL (26). From practical purposes, TC is considered normal up to ≈160 mg/dL, borderline from 170 to 199 mg/dL and high at ≥200 mg/dL (31). At variance with placebo, TC plasma reverted to the normal range (average: 149 mg/dL) as early as after 3-mo of ingesting the food supplement. On average, at enrollment, subjects had values ≤130 mg/dL. Values of 87 mg/dL (average) were achieved after 3-mo ingestion of the food supplement. Because of its potential danger to health, the LDL cholesterol limit of 130 mg/dL drops further if risk factors for cardiovascular disease (e.g., DM, overweight, hypertension, smoking, excessive alcohol consumption and a sedentary lifestyle) co-exist (31). The early significant LDL decrease observed in this report should be seriously considered in subjects that exhibit additional risk factors beyond elevated LDL cholesterol levels. Values of plasma triglycerides ≤90 mg/dL are desirable, 90–129 mg/dL are considered borderline and those ≥130 mg/dL are high. As early as after 3-mo treatment with the supplement, the mean plasma triglyceride values approached values considered optimal. Guidelines recommend HDL cholesterol values ≥40 mg/dL, optimal figures being ≥55 mg/dL for men and 65 mg/dL for women of childbearing age (31). HDL cholesterol values reached an optimal value (56 mg/dL, average) after 6 months of treatment. Regarding these beneficial effects on glucose and lipid metabolism (and, in turn the individual cardiovascular risk profile), no blood, kidney or liver indices of toxic effects were detected throughout the 6-mo observation period, in those who ingested the food supplement tested in the present study. Potential mechanisms of these effects reported here should be also considered.

Studies document the association between dietary chromium deficiency, risk factors for DM, obesity and cardiovascular disease (32). By activating intracellular signaling pathways -e.g., GLUT4 translocation, and in turn increasing glucose and amino acid transport (33), chromium is involved in carbohydrate and lipid metabolism. In clinical studies (15), chromium supplementation shows beneficial effects on indices of hyperglycemia, possibly by increasing the number of insulin receptors and the binding of insulin to its site of action (16, 34). Chromium also binds to chromodulin (LMWCr) to increase insulin receptor signaling (15, 35). In addition, chromium inhibits 3-hydroxy-3-methyl-glutaryl-CoA reductase enzyme in the liver, thus interfering with cholesterol metabolism (36). Supplementing a normal diet with chromium also improves triglyceride and HDL cholesterol levels (37). In addition, at standard doses, chromium supplementation does not increase the risk of adverse events compared to a placebo. Chromium also increases insulin-stimulated phosphorylation of several substrates, including IRS proteins (17) and phosphatidylinositol-3 kinase (PI-3 K) in skeletal muscle (38), and enhances of insulin-dependent protein kinase B (Akt), and protein tyrosine phosphatase phosphorylation (16, 39). A decrease in tyrosine phosphatase 1B (PTP1B) has also been reported, related to enhanced insulin sensitivity (17). Chromium increases the activity of 50-AMP-activated protein kinase (AMPK), suppressing a sterol regulatory element-binding protein (SREBP)-1, thus modulating the synthesis and absorption of cholesterol, triglycerides, and fatty acids (18). In addition, chromium inhibits acetyl-CoA carboxylase (ACC), thus decreasing malonyl-CoA levels, in turn increasing the oxidation and in turn the degradation of free fatty acids (40).

Together with flavan-3-ols, hesperidin, a flavanone glycoside isolated from the peels of some *Citrus* species, is present in abundance in bitter orange (*Citrus aurantium* L.) and in orange and lemon. By acting on metabolic pathways associated with glucose uptake and insulin sensitivity, flavonoids exert a positive effect in subjects at risk of type 2 DM (41). Epidemiological evidence suggests an inverse association between flavonoid intake and the incidence of type 2 DM. At variance with flavonols and isoflavones, the use of anthocyanins and flavan-3-ols reduce the risk of type 2 DM (41–43). The intimate mechanism of this beneficial effect is still a matter of investigation. The link between obesity (20), chronic inflammation and oxidative stress are well known in insulin resistance as well as in the cardiovascular risk profile (43). The modulation of insulin independent or dependent pathways is critical in the redox state and signal transmission regulating insulin secretion and synthesis in pancreatic *β*-cells; likewise, the PI3K/AKT pathway or the activation of the PPAR-*γ* receptor, modulates insulin sensitivity of peripheral districts through different tissue levels (e.g., the skeleton) (42, 43). On the other hand, the modulation of insulin independent pathways includes the activation of energy sensing molecules, e.g., AMPK in the liver, muscle and adipose tissue (44). Flavonoids actively modulate carbohydrate digestion and glucose uptake in the small intestine, through inhibition of the *α*-amylase and α-glucosidase enzyme activity and/or interference with glucose transporters (45, 46). Preclinical and clinical studies (19) show the beneficial effects of hesperidin and its aglycone (31). Both *in vitro* and in healthy volunteers, hesperidin attenuates 14C-labeled glucose transfer across differentiated Caco-2/TC7 cell monolayers (19). Fructose transport is also affected by hesperidin and in part by GLUT5 inhibition (47). Hesperidin modulates the postprandial glycemic response of orange juice by partial inhibition of intestinal GLUT, an event accounted for by sugar and hesperidin concentrations (19). In addition, hesperidin improves insulin resistance by regulating the IRS1-GLUT2 pathway via TLR4 (48). Finally, dietary hesperidin significantly improves postprandial hyperglycemia and hyperlipidemia (20). In light of the significant improvements observed also in the lipid profile, despite being a secondary endpoint, the results obtained are of relevance. Indeed, to our knowledge, this is the first clinical study investigating the effects of these specific bioactive ingredients (i.e., *Citrus limon*, *Vitis vinifera*, hesperidin, and chromium) on both glucose and lipid metabolism in subjects with impaired fasting blood glucose. The promising findings on total and LDL cholesterol, HDL, and triglycerides suggest a potential application of this supplement in subjects with borderline dyslipidemia. This is particularly noteworthy in the current context, where the need for safe and effective nutraceutical alternatives to red yeast rice is growing, due to European regulatory restrictions and safety concerns related to monacolin K content.

This randomized, double-blind, placebo-controlled trial supports the efficacy of the strategy to use the combination of different ingredients [i.e., extracts from lemon (*C. limon*) and red grapes (*V. vinifera*), hesperidin from orange (*C. sinensis*), and chromium], which act through different metabolic pathways, in individuals with impaired fasting blood glucose who are not eligible for conventional hypoglycemic therapy.

While the design of this clinical study represents a clear strength, the absence of a post-intervention follow-up limits conclusions on the long-term persistence of the observed effects. Future studies are needed to confirm these findings and to evaluate the long-term efficacy of the combination of these bioactive substances.

## 5 Conclusion

The results of this study indicate that although food ingredients alone exert mild efficacy, the combination containing extracts from lemon (*C. limon*) and red grapes (*V. vinifera*), hesperidin from orange (*C. sinensis*), and chromium, administered for 6 months to subjects with impaired fasting blood glucose, contributed to the regulation of glucose homeostasis by promoting glucose metabolism. Moreover, a beneficial effect was highlighted for lipid metabolism. Larger-scale and longer-term studies are warranted to confirm these results and to further investigate the potential role of this supplement in the management of early impaired fasting blood glucose.

## Data Availability

The original contributions presented in the study are included in the article/Supplementary material, further inquiries can be directed to the corresponding author/s.
