# Supplementary material for: Impact of a food supplement containing Citrus limon L. Osbeck and Vitis vinifera L. extracts, hesperidin and chromium in combination with an isocaloric diet on glucose and lipid metabolism in subjects with impaired fasting blood glucose: a single-center, controlled, randomized, parallel-arm, double-blind clinical trial
Source: Front Nutr. 2025 Oct 14;12:1671102. doi: 10.3389/fnut.2025.1671102 (PMC12558782; doi:10.3389/fnut.2025.1671102)
Supplement: Supplementary file 1 [file Table_1.DOCX]

Supplementary Material


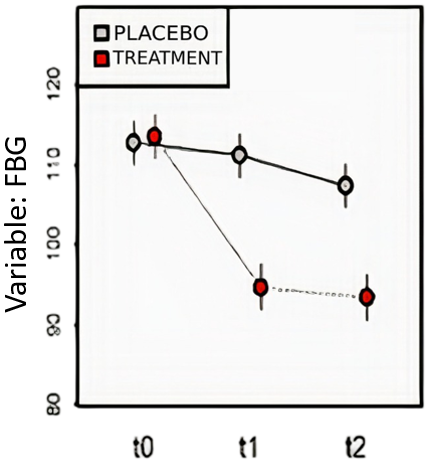


**Supplementary Figure 2**. Comparison between the supplement and placebo treatments for Fasting Blood Glucose (FBG), as predicted by linear mixed models (LMM). The analysis includes the effects of measure, treatment, sex, age, and the size × treatment interaction, with results presented as means ± 95% confidence intervals.


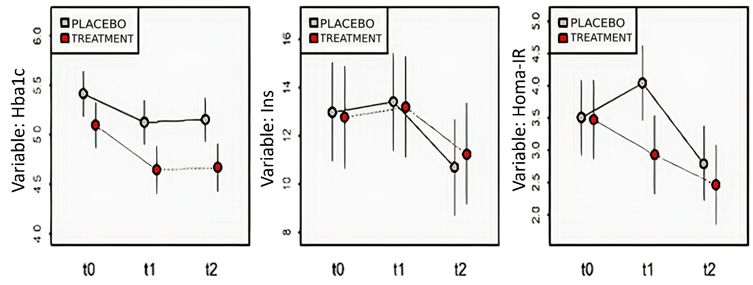


**Supplementary Figure 3.** Comparison between the supplement and placebo treatments for Glycosylated hemoglobin (HbA1c), Insulin (INS), HOMA-IR (homeostatic model assessment of insulin resistance), as predicted by linear mixed models (LMM). The analysis includes the effects of measure, treatment, sex, age, and the size × treatment interaction, with results presented as means ± 95% confidence intervals.


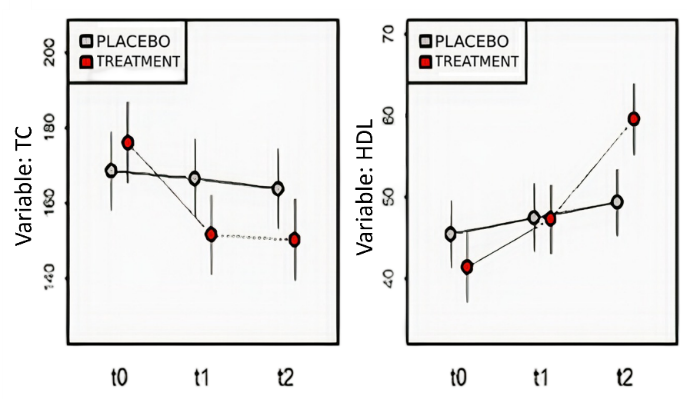


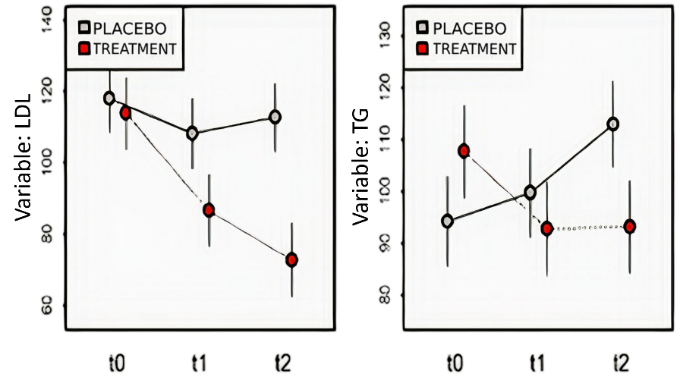


**Supplementary Figure 4**. Comparison between the supplement and placebo treatments for Total cholesterol (TC), High-density lipoprotein cholesterol, (HDL-C), Low-density lipoprotein cholesterol (LDL-C), Plasma triglycerides (TG), as predicted by linear mixed models (LMM). The analysis includes the effects of measure, treatment, sex, age, and the size × treatment interaction, with results presented as means ± 95% confidence intervals.


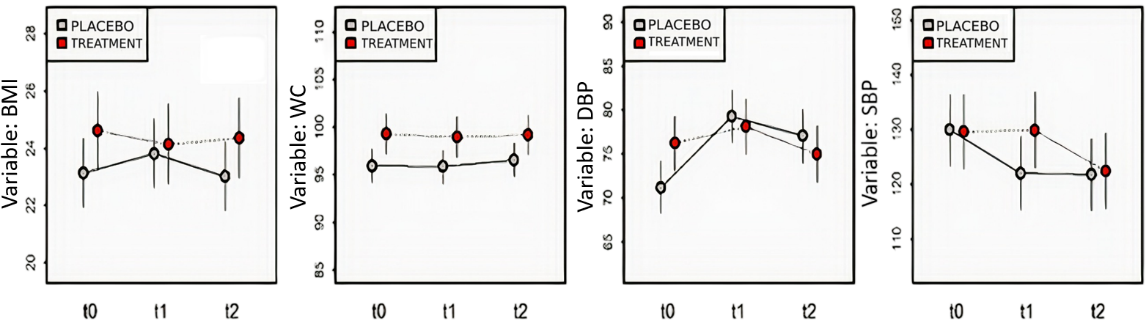


**Supplementary Figure 5**. Comparison between the supplement and placebo treatments for Body mass index (BMI), Waist circumference (WC), Diastolic blood pressure (DBP), Systolic blood pressure (SBP), as predicted by linear mixed models (LMM). The analysis includes the effects of measure, treatment, sex, age, and the size × treatment interaction, with results presented as means ± 95% confidence intervals.


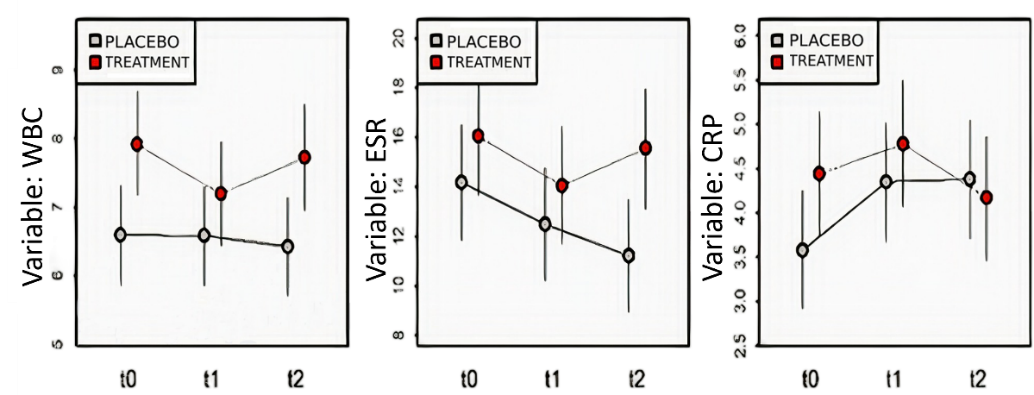


**Supplementary Figure 6**. Comparison between the supplement and placebo treatments for White blood cell counts (WBC), Erythrocyte sedimentation (ERS), C-reactive protein (CRP), as predicted by linear mixed models (LMM). The analysis includes the effects of measure, treatment, sex, age, and the size × treatment interaction, with results presented as means ± 95% confidence intervals.

*
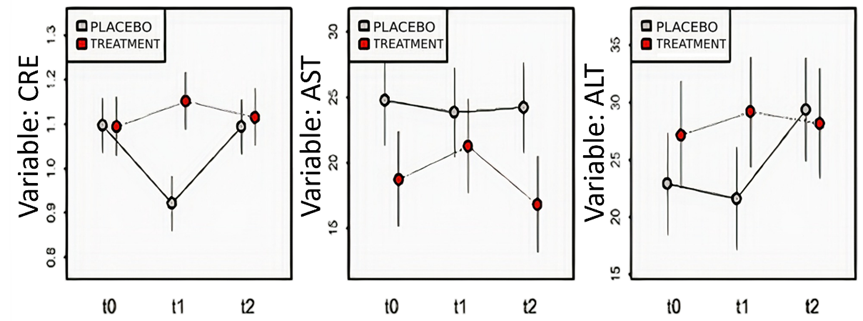
*

**Supplementary** **Figure 7**. Comparison between the supplement and placebo treatments for Creatinine (CRE), Aspartate transaminase (AST), Alanine transaminase (ALT) and other related variables, as predicted by linear mixed models (LMM). The analysis includes the effects of measure, treatment, sex, age, and the size × treatment interaction, with results presented as means ± 95% confidence intervals.
